# Interprofessional peer-assisted learning as a low-threshold course for joint learning: Evaluation results of the interTUT Project

**DOI:** 10.3205/zma001029

**Published:** 2016-04-29

**Authors:** Kathrin Reichel, Stefan Dietsche, Henrike Hölzer, Michael Ewers

**Affiliations:** 1 Charité - Universitätsmedizin Berlin, Department for Curriculum Management, Berlin, Germany; 2 Charité – Universitätsmedizin Berlin, Institute of Health and Nursing Science, Berlin, Germany; 3 Alice Salomon University of Applied Sciences, Degree Course Physical/Occupational Therapy, Berlin, Germany

**Keywords:** Interprofessional Education, Peer-Assisted Learning, Interprofessional Relations, Student Learning

## Abstract

**Background and objective: **The delivery of needs-based health care services requires a team-based and collaborative approach of different health professionals, which is not yet sufficienctliy implemented on a day to day basis. Interprofessional learning activities aim to respond to this in future. The cross-university pilot project interTUT used peer-assisted learning approaches and extracurricular tutorials in order address this issue.

**Methodology: **During the pilot phase, eight students and trainees have been acquired. Together, they prepared and led four extracurricular tutorials on core topics of interprofessional cooperation and documented them in procedure manuals. The course was evaluated using a standardized participant survey (n=72) and two focus groups (n=3, n=5) in which participants were asked to reflect on their individual learning experiences. Descriptive statistics were used to analyze the survey data and the focus group material was interpreted using qualitative content analysis.

**Results: **The results indicated a high level of satisfaction, acceptance of and further demand for peer-supported learning activities. The students and trainees reported changed attitudes and subjective knowledge growth regarding the other professional groups. The constructive learning atmosphere as well as having access to a forum for interprofessional exchange were equally valued.

**Conclusions: **Extracurricular tutorials offer a low-threshold and very promising point of contact for the facilitation of interprofessional teaching and learning. However, this should be viewed against the background that, as part of the pilot project, only a small number of students and trainees who were already interested in the topic could be reached by this optional course. A comprehensive, long-term trial of this teaching and learning format, its linkage to curricular courses, and further research on its education-specific and practice-related effects are, therefore, necessary.

## Zusammenfassung

**Hintergrund und Zielsetzung:** Die für eine bedarfsgerechte Gesundheitsversorgung erforderliche Kooperation unterschiedlicher Gesundheitsprofessionen will im Alltag noch selten gelingen. Dem soll künftig durch Angebote interprofessionellen Lernens und Lehrens begegnet werden. Hierfür Ansätze des Peer-Assisted Learning aufzugreifen und extra-curriculare Tutorien nutzbar zu machen, war Ziel des hochschulübergreifend angelegten Pilotprojekts interTUT.

**Methodik: **In der Pilotphase konnten acht Studierende und Auszubildende gewonnen werden, die gemeinsam vier extra-curriculare Tutorien zu Kernthemen interprofessioneller Zusammenarbeit entwickelt, durchgeführt und in Form von Ablaufmanualen dokumentiert haben. Die Evaluation erfolgte in Form einer standardisierten und deskriptiv-statistisch ausgewerteten Teilnehmerbefragung (n=72) sowie zweier inhaltsanalytisch ausgewerteter Fokusgruppen (n=3, n=5) zur Reflexion individueller Lernerfahrungen.

**Ergebnisse:** Die Evaluationsergebnisse lassen eine hohe Zufriedenheit, Akzeptanz und weiteren Bedarf an peer-gestützten Lernangeboten erkennen. Die Studierenden / Auszubildenden berichten über veränderte Einstellungen und subjektiven Wissenszuwachs über die anderen Berufsgruppen. Die konstruktive Lernatmosphäre wird ebenso geschätzt wie das Forum zum interprofessionellen Austausch.

**Schlussfolgerung: **Extra-curriculare Tutorien bieten einen niedrigschwelligen und viel versprechenden Anknüpfungspunkt zur Förderung interprofessionellen Lehrens und Lernens. Dabei ist zu bedenken, dass im Rahmen des Pilotprojekts bislang nur wenige und bereits am Thema interessierte Studierende/Auszubildende mit diesem freiwilligen Angebot erreicht werden konnten. Notwendig ist eine umfassendere und längerfristig angelegte Erprobung dieses Lehr-Lernformats, eine Verschränkung mit curricularen Lehr-Lernangeboten sowie weitere Forschung zum bildungsspezifischen Nutzen und praxisbezogenen Effekten.

## 1. Introduction and background

The provision of healthcare, which is becoming ever more complex, calls for interprofessional collaboration in all healthcare settings. Such collaboration can contribute to improving the quality of healthcare, as well as patients’ satisfaction and quality of life [1], [2], [3], [4], shortening hospitalization periods, reducing costs and improving healthcare professionals’ job satisfaction [5]. However, lack of information, stereotypes, conflicts of interest and inflexible regulations and surrounding conditions hinder interprofessional collaboration. To counter this, numerous initiatives have been developed on an international level, including the implementation of interprofessional learning and teaching as early as possible in health professions` education [http://caipe.org.uk/ cited 2015 Jul 15], [http://whqlibdoc.who.int/hq/2010/WHO_HRH_HPN_10.3_eng.pdf cited 2015 Jun 9]. Currently available research findings on such initiatives illustrate their positive effects on students’ satisfaction, attitudes and skills [1], [6], [7]. At the same time, it is currently more difficult to prove the long-term results of interprofessional teaching and learning, and, in particular, its effects on healthcare practice [8].

By now, the topic has been recognized as a pressing concern in Germany [9], [10], [11], [12]. However, healthcare professionals within this country are trained at different levels (undergraduate course, vocational trainingschools), at different learning facilities (full university, university of applied sciences, vocational schools) and according to heterogeneous guidelines (regulations on licensing, different traineeship and examination ordinances) [13], [14], [15], [16]. The introduction of interprofessional learning and teaching courses has, therefore, been set a number of cultural, curricular, logistic and legal hurdles. To address these in a creative way, the Robert Bosch Foundation has introduced the support program “Operation Team”.

## 2. Project description: interTUT – an interprofessional teaching and learning course

### 2.1. Objectives, organization and conception

The interTUT pilot project was fostered within the context of this program from 2013 to 2015. The acronym stands for *inter*professional *tut*orials and is, therefore, an optional extracurricular teaching and learning activity at which students (tutors) and their fellow students (tutees) instruct and support each other in learning together. The objective of the project was to develop and test cross-professional tutorials on different topics with students and trainees from different undergraduate and vocational training courses (medicine, physical and occupational therapy, nursing) from three different faculties in Berlin.

The project was coordinated at the Charité– Universitätsmedizin Berlin together with the vice dean for teaching, represented by the Department for Curriculum Management, with the learning center and the simulated patient program, and the Institute of Health and Nursing Science. The Charité Health Academy, which provides vocational training in nursing, and the Alice Salomon University Berlin, which offers an undergraduate course in physical therapy and occupational therapy, were also partners. The pilot project brought together different faculties, programs and levels of education. 

Two basic concepts guided interTUT: Firstly, an extracurricular optional course was consciously adopted which made a considerable number of experiences available. Numerous peer-teaching tutorials on different, mostly self-chosen, topics have been developed alongside the compulsory curriculum for and by medical students at the learning center of the Charité [http://aco.charite.de/studierende/lernzentrum/tutorien/ cited 2015 June 22]. These voluntary learning activities were to be made available within the context of interTUT for other healthcare professionals and utilized for learning “from, with and about each other.”, referring to the definition of interprofessional education by the British Centre For The Advancement of Interprofessional Education (CAIPE) in 2002.

Simultaneously, the advantages of this kind of peer-assisted learning format were to also be developed for the training of healthcare professionals other than doctors. In Germany, in comparison to medicine [17], [18], [19], tutorials are seldom utilized in vocational training or in new degree programs for nursing, physical or occupational therapy. This optional interprofessional teaching and learning course was to be implemented with the appropriate level of success, without great expenditure, quickly and sustainably at other university locations. 

The second conceptual element of interTUT was its concentration on peer-assisted learning (PAL) approaches. An expanded definition from Topping (1996) may elucidate this: “People from similar social groupings who are not professional teachers helping each other to learn and learning themselves by teaching” [20]. Students took up different roles as teachers and students in non-hierarchical teaching and learning environments within the framework of this program. Some of the advantages for students, among others, are better performance and examination results, a higher level of satisfaction with their studies and a reduction in drop-out rates [17], [21]. The PAL approach supports communal learning as opposed to competitive learning, and so helps to improve social cohesion. This is also what makes interprofessional learning interesting. In addition, PAL fosters active, independent, self-directed learning, and enables peers to follow their own agenda and fill self-identified “gaps” in the curriculum. By taking over the role of tutor, competencies and future roles in teamwork were tested out at the same time. Using this potential for interprofessional learning was a further priority of interTUT.

#### 2.2. Development and testing of interprofessional tutorials

Within the context of the project, four in topic different interprofessional tutorials were to be developed and tested. Guiding principles included best practice recommendations for interprofessional learning, such as small-group work, practiced-based learning, patient scenarios or simulation and learning units of at least 2.5 hours or longer [22], [6].

Firstly, a cross-university, cross-institutional and inter-professional project team with representatives from the participating project partners was established (see above). The task of this committee and for the project workers responsible for the implementation of the tutorials was the development of a basic concept, project management and support for the tutor team. The development of the tutorial content was the task of the students and trainees from the participating faculties. An open collection of topics was carried out over the course of two workshops, which were subsequently reduced and substantiated by cluster methods. As a result of this, a high content overlap for interprofessional learning was found with regards to the internationally applicable general recommendations [http://whqlibdoc.who.int/hq/2010/WHO_HRH_HPN_10.3_eng.pdf cited 2015 Jun 9], [23], [24].

Students in medicine, occupational therapy, physical therapy and nursing were employed as undergraduate assistants to take over as tutors, in coordination with the project partners, for the structuring of the content and implementation of the tutorials. A prerequisite for this role was that the students had to be enrolled in an undergraduate program or vocational training at one of the participating and cooperating institutions, and that they had to be studying in an advanced semester. Selection criteria included previous experience in teaching, group work or civic engagement, an interest in working with students and interprofessional learning, organizational skills, the ability to work in a team and flexibility.

The qualification of these tutors was based on different relevant recommendations [18], [19], [25]. In practice, basic training of up to eight hours was carried out (e.g. on facilitation techniques, teamwork procedures, the role of the tutor) which was expanded through further training courses as required (e.g. on simulated patients in teaching). In addition, experienced tutors were able to act as “observational colleagues” at the learning center. Over the duration of the project, a total of eight tutors became qualified; three each from medicine and nursing, and one student each from physical and occupational therapy.

The interprofessional tutorials were offered as involving team teaching by tutors from at least two different training courses. Advertising for the extracurricular course was carried out independently by the tutors (mailing lists, posters, personal appearances at lectures and posts on social networks). The individual learning units were developed one after the other; individual sequences were then tested in interprofessional team-teaching and eventually carried out as a pilot course with participants, and revised if necessary according to feedback from the participants and tutors.

Table 1 (Tab. 1) shows an overview of the four learning units. Previous knowledge was recommended for the tutorials based on clinical skills; however, in general, a particular level of training was not required for the courses. The relevant manuals have not yet been published and are still in the preparatory stages.

#### 2.3. Objectives and methods of evaluation

Based on the project concept, the aim of the evaluation was to identify the effective elements of and challenges to these interprofessional tutorials. Additionally, goal attainment from the perspective of the participants was to be evaluated, e.g. knowledge on roles and tasks, competency gains of the tutees and tutors, and assessment of the learning environment in the tutorials. A standardized continuous participant survey was employed methodically, as well as additional focus groups. 

The standardized participant survey took place at the end of each tutorial in the form of a written evaluation on paper. An evaluation form which had already been utilized in tutorials for medical students at the learning center of Charité, was expanded thematically and used for the optional courses [http://aco.charite.de/lehrende/lernzentrum/tutorien/evaluation_der_tutorien/ cited 2015 November 15]. This was comprised of 25 questions, e.g. on the organization, scope and content of the tutorial, whether the course was too challenging or not challenging enough, and evaluation of the tutorial for the respective level of training and later professional life. A uniform answer format from “fully agree” to “fully disagree” on a 7-level scale was provided for the closed-ended questions. The open-ended questions were directed towards the learning atmosphere, the subjective learning gains and further thematic suggestions. The evaluation carried out was descriptive-statistic in nature. 

After all four tutorials had been developed and a sufficient basic level of heterogeneity of participants had been reached, two focus groups of 60 – 90 minutes composed of different professions, independent of the tutorial appointments, were carried out, one with the tutors and one with the tutees, respectively. The latter was formed using an anonymous participant mailing list. Participation was voluntary for both groups. What was interesting in the focus groups was the specific and general learning effect of interprofessional tutorials from the viewpoint of the participant and how this could be improved. The evaluation analyzing the content of data was deductive–inductive [26]. Our expectations [27] for the description of the learning gains were based on the IPE hierarchy of outcome levels according to Kirkpatrick, modified Barr et al [28].

## 3. Results: Evaluation of interprofessional tutorials

**Supply and demand: **30 tutorial dates were offered between June 2014 and June 2015, of which 12 tutorials took place. A total of 74 people took part. The tutorials were developed and offered one after the other. Tutorial 1 took place six times, Tutorial 2 thrice, Tutorial 3 once and Tutorial 4 twice. The first challenge was “testing” or identifying a time slots when the most people could attend. As a result of this, the tutorials were held twice a month on a Friday or a Saturday in addition to regular classes, reduced during the examination and holiday periods of February/March and August/September. 

**Description of the sample:** Table 2 (Tab. 2) shows the distribution of participants from undergraduate and vocational training programs and their semester on average. For a tutorial to be held, applications had to be submitted from at least two professional groups and by at least five participants. Having all professions present at all course sessions was not possible; however, at six meetings at least three training courses were represented; in the others, at least two. On average, the participants were in their fourth semester, medical students in their first training phase, and the others in the second half of their education.

**Figure 1:**
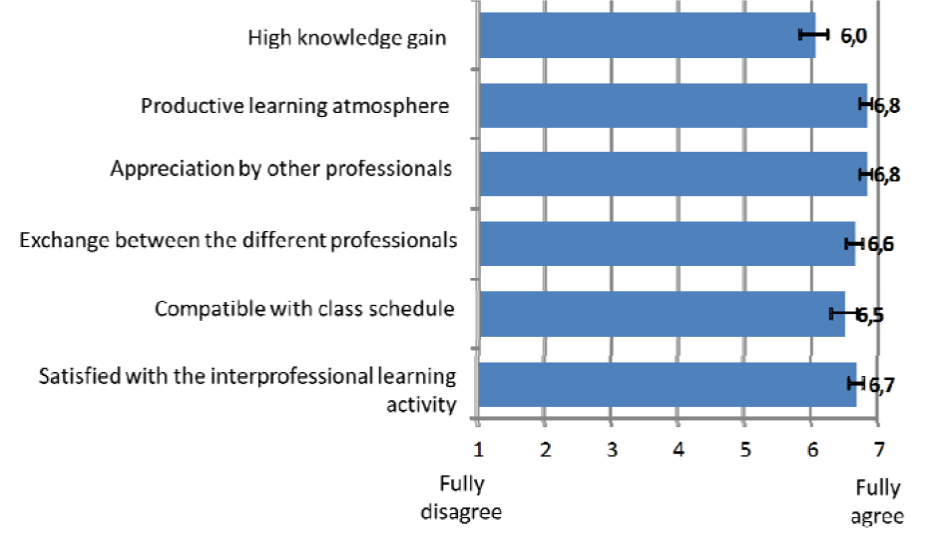
Rating of the interprofessional tutorials (n=72; mean average and 95% confidence interval)

**Results of the written survey: **The main results of the standardized participant survey can be taken from Figure 1 (Fig. 1). The feedback rate was 97 % (n=72 out of 74). It shows that the assessments were generally positive. The participants reported a very productive learning environment. The exchange of information between professional groupings was facilitated by the tutorials; correspondingly, a mutually respectful atmosphere was reported. Overall, the course was also perceived as compatible with the timetable; however, this, of course, only related to people who were able to attend. The positive overall assessment showed a very high level of overall satisfaction (6.7 points); however, most of the subjectively-perceived learning progress lagged behind the other values. 

The level of knowledge required for the tutorials for the overwhelming proportion of participants was estimated as the following: 81% (n=58) of the participants reported that they felt neither over-challenged or under-challenged, the rest, 19 % (n=14), admitted that they felt “somewhat under-challenged” as opposed to “under-challenged”. 

The students and trainees felt welcome at the learning center and felt that the content was very important for their education and their later professional life. In the comments, the participants emphasized the open exchange of information between the participating professional groups; they particularly liked the learning atmosphere and the practical content. Suggestions for improvements included primarily the call for more of an overview of the activities of the individual professional groups and the inclusion of more professional groups.

**Results from the focus groups: **Results from the focus groups: Participants from three different professional programs were represented in both focus groups with tutors (n=3) and with tutees (n=5). The discussion sessions were spread out over about 60 – 90 minutes, based on key issues developed previously [29] and were both intensive and comprehensive. The strengths and weaknesses of the tutorials and the subjective learning gains were discussed. The provisional analysis shows that both tutors and tutees observed a change in their attitude and perception. The increase in knowledge of the respective tasks, their self-image and the different perspectives on common practice were valued by the participants. The learning atmosphere, characterized by openness and understanding as a “space for common exchange,” was deemed important, or, in the words of one participant, “(...) it was great that such a platform, where you could simply just exchange information, existed” (FG1). This exchange, among others, was made possible by the small group format and the application of PAL, through which the usual pedagogical issues in classes could be avoided, as demonstrated by one participant’s statement: “Well, I think, if a professor had been standing there, (...) I would have felt tense again on the inside” (FG2). The results of the written survey and the focus groups proved to be generally consistent. The answers to the questions on satisfaction, potential for improvement, the subjectively perceived increase in knowledge and the positive learning atmosphere were simply expanded upon.

## 4. Discussion

Following on from previous experiences with extracurricular tutorials in university medicine, cross-organizational interprofessional teaching and learning experiences could be facilitated with interTUT without having to factor in laborious curriculum reforms or structural changes.

The level of satisfaction with the individual tutorials was exceptionally high. Perhaps this finding may be relativized by the fact that because the students and trainees were already interested in interprofessional learning, they evaluated the voluntary course more positively due to of their own inclinations. Other extracurricular tutorials at the learning center were also evaluated positively compared to curricular teaching. The quality of PAL optional courses was, however, not questioned as a result. The acceptance of interTUT tutorials may be viewed as proof that students and trainees show an interest in the topic, despite such things as additional workloads and unfavorable class times. 

Particularly because of its extracurricular character, interTUT proved itself to be easily implementable and also applicable to other educational contexts (e.g. the new undergraduate programs in nursing and allied health professions). This is also valuable in international contexts. However, the resources for tutors, their qualification and the coordination of optional courses will have to be taken into consideration. In addition, these types of optional learning and teaching opportunities require the willingness to collaborate fully on a higher level with university and professional traineeship facilities, undergraduate and vocational training programs, and representatives employed within different professions. This succeeded in the project at hand for the teachers, as well as the students; however, it may not be possible in every context, and additionally, it is time and resource intensive and has many prerequisites.

The interprofessional tutorials based on PAL were well received by the students and valued as a meeting space outside of the hierarchical structures within and between the professions. Similarly positive experiences are being reported with the interprofessional application of PAL in London [30]; in general, however, it is still only used very rarely. Better utilization of the potential of PAL for interprofessional teaching and learning is a highly promising path.

The overwhelmingly positive evaluation results must be emphasized as they relate to the interprofessional character of the tutorials; the productive learning environment, the high regard for other professional groups and the fostering of interprofessional exchange are all part of this. It seems that these stem from the qualified team of tutors and the importance of team teaching. At the same time, this finding may be seen as a validation of the tutorial content and, simultaneously, proves the recommendation that interprofessional learning should be offered in smaller groups when possible [22].

On average, two to three professional groups were represented in the tutorials, which allows one to assume that not all classes were organizationally compatible with all four educational programs. Whether the optional course would prove itself easier to implement with only two to three professional groups must, therefore, be examined. It is also possible that different curricular requirements and the high workloads of students and trainees due to their mandatory curricular courses represent organizational barriers. This indicates that curricular credit transfer options for interTUT tutorials should probably be developed.

A further concern for the future of interprofessional learning is that we were only able to attract students and trainees who had time alongside their other obligations and an already pronounced interest in interprofessional collaboration; interTUT shares these weaknesses with other voluntary initiatives (e.g. action days, introductory weeks). Targeted and persuasive advertising campaigns are also required within the curricular learning processes. In addition, learning “from each other, with each other and through each other” (referring to the CAIPE definition of IPE 2002) is also a significant goal of this teaching and learning format. Despite the high levels of satisfaction, the rather limited increase in knowledge of the respective themes and the proportion of rather under-challenged tutees are important, in hindsight, and demand a more intensive analysis of the level of tutorial content. Apparently, the added value of the tutorials decreases with an increase of knowledge, but they were seen instead as more of a “space for common exchange,” as the evaluation results suggest. The learning results would, therefore, be related to mindsets and attitudes instead. Finally, the tutorials should not remain the single optional course; they should alternately interlock closely with curricular teaching and learning options on the topic and be expanded upon with practical interprofessional experiences. There is a need for further development in this area. 

## 5. Conclusions

Providing a low-threshold option for interprofessional teaching and learning is just as much an important task as it is a difficult one. In the case of the pilot project interTUT, an optional peer-supported teaching and learning course was highly successful overall. Within a limited period of time, an important meeting space could be created and an impulse for interprofessional teaching and learning was generated, even in the early phases of professional training. To be able to gain further results from this optional PAL course, for example, on their education-specific value or their effects on medical practice, it must be tested out in the future over a longer period of time, with a larger number of participants, and, if possible, at multiple centers. Committed educational providers, sufficient resources, an exchange of experience on an international level, and more research on interprofessional teaching and learning are therefore necessary.

## Funding

The project has received a grant from the Robert Bosch Stiftung (project number 32.5.1316.0006.0).

## Acknowledgements

Further thanks given to the project group, all the students who participated, our colleagues in the Department of Curriculum Management and the Institute of Health and Nursing Science for their involvement in the project, as well as our colleagues from the evaluation department for the questionnaire data administration.

## Competing interests

The authors declare that they have no competing interests.

## Figures and Tables

**Table 1:**
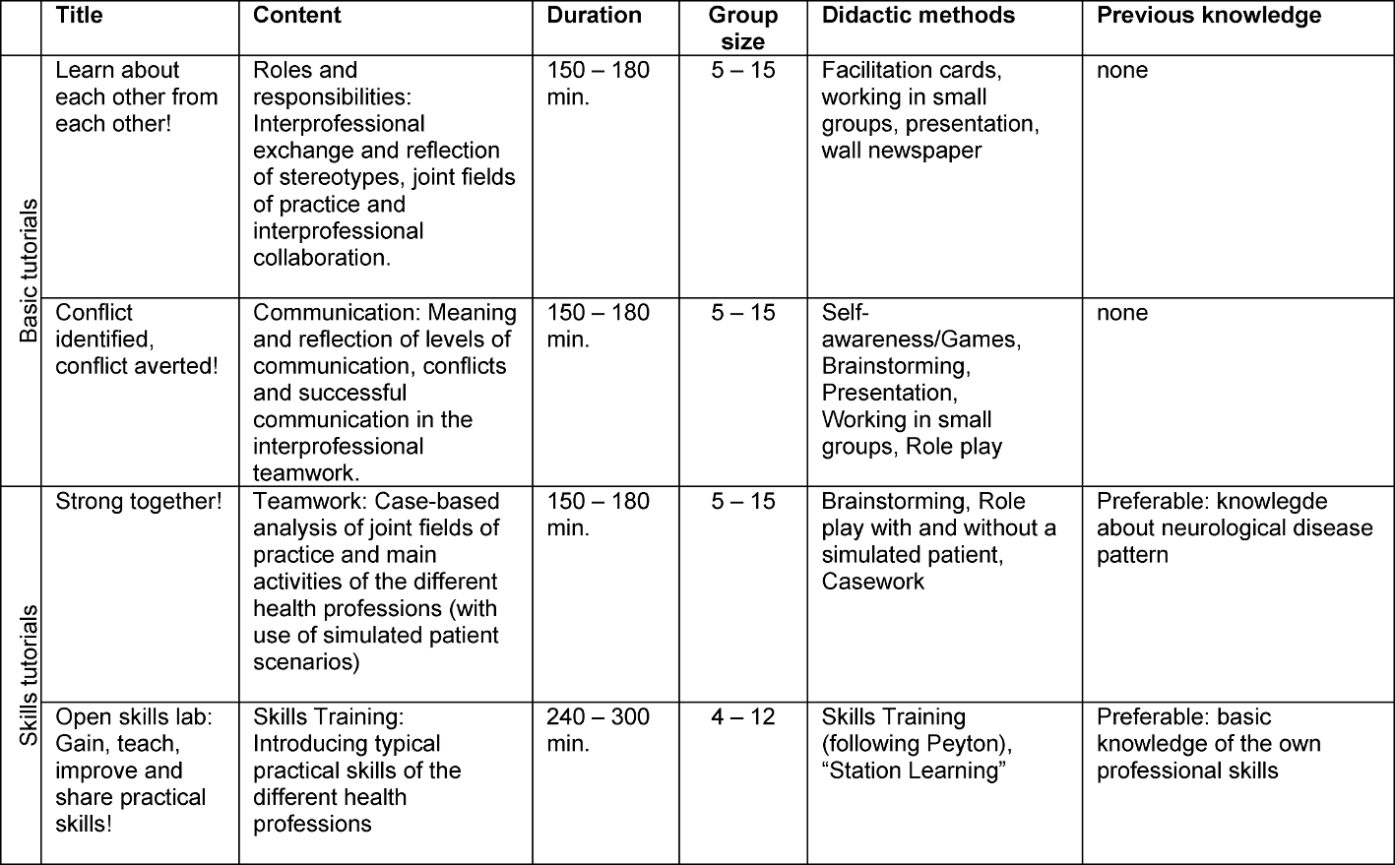
Interprofessional interTUT tutorials in brief

**Table 2:**
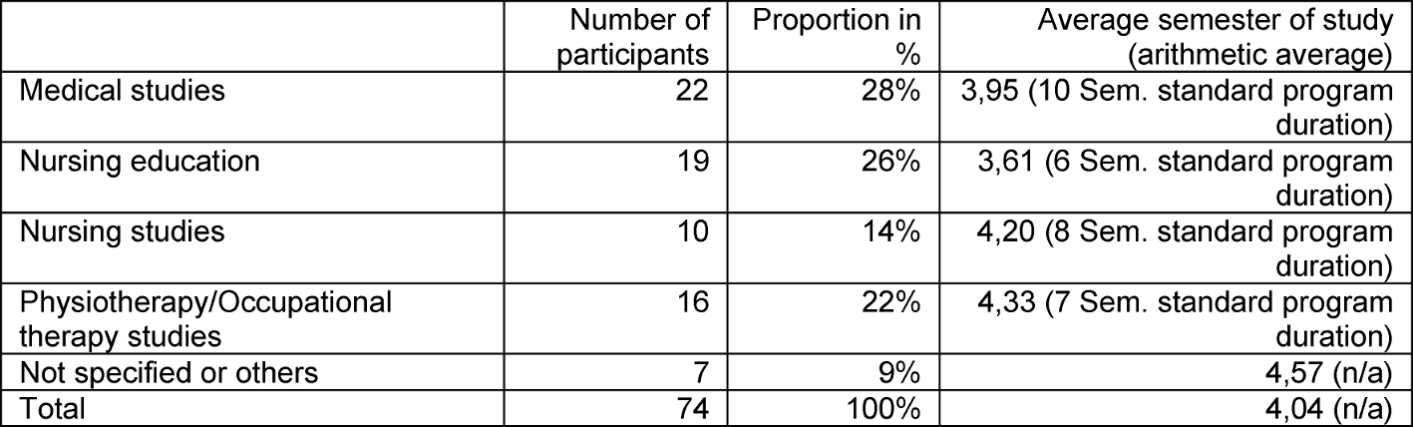
Sample description
